# Investigation of a Pharmacological Approach for Reduction of Renal Uptake of Radiolabeled ADAPT Scaffold Protein

**DOI:** 10.3390/molecules25194448

**Published:** 2020-09-28

**Authors:** Anzhelika Vorobyeva, Maryam Oroujeni, Sarah Lindbo, Sophia Hober, Tianqi Xu, Yongsheng Liu, Sara S. Rinne, Javad Garousi

**Affiliations:** 1 Department of Immunology, Genetics and Pathology, Uppsala University, 751 85 Uppsala, Sweden; anzhelika.vorobyeva@igp.uu.se (A.V.); maryam.oroujeni@igp.uu.se (M.O.); tianqi.xu@igp.uu.se (T.X.); Yongsheng.Liu.6513@student.uu.se (Y.L.); 2 Research Centrum for Oncotheranostics, Research School of Chemistry and Applied Biomedical Sciences, National Research Tomsk Polytechnic University, 634 050 Tomsk, Russia; 3 Department of Protein Technology, KTH—Royal Institute of Technology, 10691 Stockholm, Sweden; slindbo@kth.se (S.L.); sophia@kth.se (S.H.); 4 Department of Medicinal Chemistry, Uppsala University, 751 23 Uppsala, Sweden; sara.rinne@ilk.uu.se

**Keywords:** kidney, reabsorption, renal uptake, radionuclide, ADAPT

## Abstract

Albumin binding domain-Derived Affinity ProTeins (ADAPTs) are small (5 kDa) engineered scaffold proteins that are promising targeting agents for radionuclide-based imaging. A recent clinical study has demonstrated that radiolabeled ADAPTs can efficiently visualize human epidermal growth factor receptor 2 (HER2) expression in breast cancer using SPECT imaging. However, the use of ADAPTs directly labeled with radiometals for targeted radionuclide therapy is limited by their high reabsorption and prolonged retention of activity in kidneys. In this study, we investigated whether a co-injection of lysine or gelofusin, commonly used for reduction of renal uptake of radiolabeled peptides in clinics, would reduce the renal uptake of [^99m^Tc]Tc(CO)_3_-ADAPT6 in NMRI mice. In order to better understand the mechanism behind the reabsorption of [^99m^Tc]Tc(CO)_3_-ADAPT6, we included several compounds that act on various parts of the reabsorption system in kidneys. Administration of gelofusine, lysine, probenecid, furosemide, mannitol, or colchicine did not change the uptake of [^99m^Tc]Tc(CO)_3_-ADAPT6 in kidneys. Sodium maleate reduced the uptake of [^99m^Tc]Tc(CO)_3_-ADAPT6 to ca. 25% of the uptake in the control, a high dose of fructose (50 mmol/kg) reduced the uptake by ca. two-fold. However, a lower dose (20 mmol/kg) had no effect. These results indicate that common clinical strategies are not effective for reduction of kidney uptake of [^99m^Tc]Tc(CO)_3_-ADAPT6 and that other strategies for reduction of activity uptake or retention in kidneys should be investigated for ADAPT6.

## 1. Introduction

Targeting aberrant overexpression of molecular targets on cancer cells is an efficient strategy to deliver radioactivity to tumors and reduce damage to normal tissues. Previous studies suggest that there is a relationship between tumor uptake, size of a targeting agent, and its affinity [1,2,3]. Small targeting molecules have high accumulation in tumors at early time points. However, for prolonged tumor retention, high affinity is required [4]. Small engineered scaffold proteins (ESPs) with the size of 4–20 kDa bind to selected antigens with high specificity and affinity [5,6,7]. The small size of ESPs is beneficial for their use in radionuclide diagnostic imaging as it allows imaging on the day of injection and minimizes the false positive tumor diagnosis due to enhanced permeability and retention effect, commonly observed for monoclonal antibodies. Clinical studies using two types of ESPs, affibody molecules and Albumin Binding Domain (ABD)-Derived Affinity Proteins (ADAPTs), confirmed their superior specificity and sensitivity for radionuclide molecular imaging of human epidermal growth factor receptor 2 (HER2) [8,9,10]. Other ESPs, such as cystine knot miniproteins and designed ankyrin repeat proteins (DARPins), demonstrated promising results in preclinical studies [11,12,13,14,15,16]. ADAPTs, novel small scaffold proteins (5 kDa) consisting of 46 amino acids, have been developed from an engineered albumin-binding domain (ABD) [17]. Three-helical structure of ADAPTs is independent of disulfide bridges and easily refolds after thermal or chemical alteration [18]. Randomization of 13 amino acids in the ABD structure created a library that includes a variety of binders for different antigens of interest. Bispecific binders with binding to tumor necrosis factor (TNFα), HER2 or HER3 from one side of ADAPT scaffold and to albumin from another side were selected [18,19]. One bispecific ABD binder that lost binding to albumin after re-engineering was called ADAPT [19]. Anti-HER2 variant with 1 nM affinity to HER2, no measurable binding to albumin, and rapid blood clearance was termed ADAPT6. This variant was used for imaging of HER2-expressing ovarian carcinoma SKOV-3 xenografts in nude mice and 1 h after injection could visualize the tumors with high contrast [17]. Different aspects of molecular design of ADAPTs for imaging and targeted therapy applications were investigated and the most promising variant of ADAPT was recently evaluated for SPECT imaging of HER2 expression in breast cancer in a clinical study [20,21,22,23,24,25].

The main excretion pathway for proteins below 60 kDa is via kidneys. After filtration through kidney glomeruli, small radiolabeled proteins are efficiently reabsorbed in proximal tubules. In tubular cells, radiolabeled ESPs are degraded with formation of radiometabolites. Residualizing radiometabolites are typically formed after degradation of proteins labeled with radiometals (e.g., lutetium-177) and are retained inside kidney cells for a prolonged period of time. Since the size of ADAPTs (5 kDa) is below the kidney filtration barrier, they are readily filtered through the glomerulus and are efficiently reabsorbed in proximal tubules [17,21]. Targeted therapy using ADAPTs labeled with radiometals is promising. However, the reduction of renal uptake of activity is necessary. Accumulation of radioactivity in kidneys during radionuclide therapy is undesirable, as it might result in nephrotoxicity and loss of function.

One strategy to reduce the retention of activity in kidneys is to use non-residualizing labels (e.g., radioiodine). After the uptake and degradation of a radiolabeled protein inside the cells, radiometabolites of non-residualizing labels diffuse through cell membranes to the extracellular space and eventually return back to blood circulation. Earlier we have investigated the influence of the residualizing effect of the label on biodistribution properties of ADAPT6. Low retention of [^131^I]I-HPEM-ADAPT6 in kidneys provided a precondition for radionuclide targeted therapy of HER2-positive tumors [21]. However, we have found that the tumor retention of ADAPT6 labeled with a radiometal was higher compared with the radiohalogen [23]. Furthermore, reduction of renal uptake using the non-residualizing label strategy is not applicable for clinically important radiometals, such as lutetium-177 and actinium-225.

An alternative strategy for reduction of renal uptake of scaffold protein-based targeting agents is fusion to ABD to increase the size above 60 kDa barrier in kidneys. Tolmachev et al., have applied this strategy to anti-HER2 affibody molecules and demonstrated that the ABD-fused affibody molecule labeled with lutetium-177 had 25-fold lower kidney uptake of activity compared to the non-fused affibody [26]. A single injection of 17–22 MBq of [^177^Lu]Lu-CHX-A″-DTPA-ABD-(Z_HER2:342_)_2_ effectively prevented the formation of tumors in mice bearing HER2-expressing xenografts [26].

Pretargeting is another approach to reduce renal accumulation of activity. In this strategy, targeting takes place in several steps. The primary targeting agent (e.g., antibody or ESP conjugated to a recognition tag) is administered first, and the delivery of the radionuclide conjugated to the secondary recognition tag is achieved in the second step [27,28,29]. In the gap between two deliveries, the primary agent is cleared from blood and cell membranes in normal organs and tissues but not from tumors. Afterwards, the secondary agent is administered that binds with high affinity to the primary agent present mainly on the membranes of tumor cells. Westerlund et al., demonstrated that the dose delivered to the tumor in affibody-based pretargeting, mediated by peptide nucleic acid (PNA) hybridization, was 5-fold higher than the dose to the kidneys [29]. This approach provided an efficient reduction of tumor growth and extension of survival of mice bearing SKOV-3 xenografts without causing any pathological changes in kidneys [29].

The use of pharmacological agents that prevent or reduce renal uptake of radiolabeled proteins might be another option that does not require modification of the targeting agent. Previously this approach has been investigated for reduction of renal uptake of several protein-based radiopharmaceuticals, such as somatostatin analogs [30,31,32]. However, the effect of these agents on renal uptake might be different for different ESPs and cannot be predicted beforehand.

We have recently studied the effect of several pharmacological agents on the renal uptake of radiolabeled DARPins [33] and affibody molecules [34]. In the present study, we continue to investigate the effect of several compounds (fructose, sodium maleate, colchicine, mannitol), drugs (probenecid, furosemide) and agents used in clinics (gelofusine, lysine) for prevention of kidney uptake of another class of ESPs, ADAPTs.

## 2. Results

**Figure 1:**
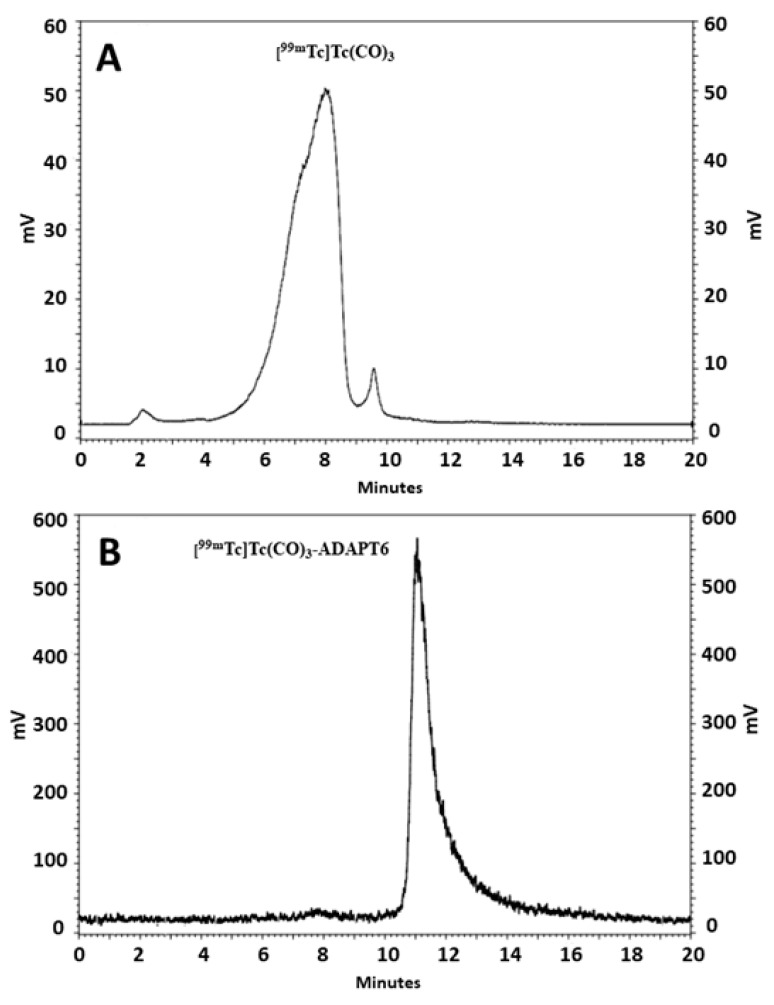
Reversed-phase high performance liquid chromatography (RP-HPLC) analysis of [^99m^Tc]Tc(CO)_3_ (**A**) and ADAPT6 labeled with [^99m^Tc]Tc(CO)_3_ (**B**).

ADAPT variant carrying a hexahistidine-tag at C-terminus, termed herein ADAPT6, was site-specifically radiolabeled with [^99m^Tc]Tc(CO)_3_ to form a residualizing label. ADAPT6 was radiolabeled with [^99m^Tc]Tc(CO)_3_ with 92.5  ±  1.5% radiochemical yield and purified to provide a radiochemical purity over 99% (Figure 1).

**Figure 2:**
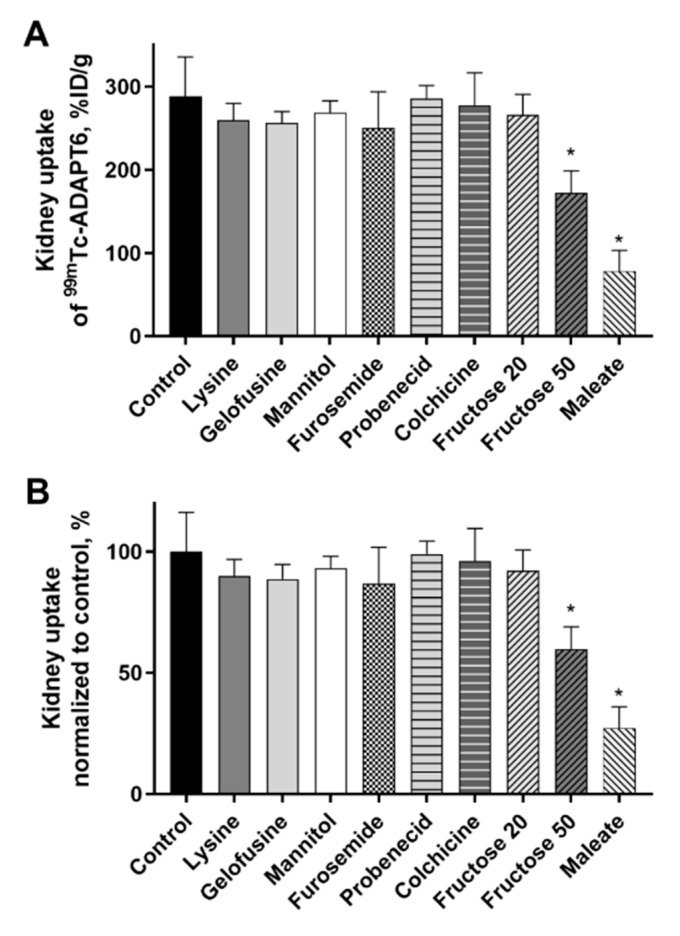
Kidney uptake of radiolabeled [^99m^Tc]Tc(CO)_3_-ADAPT6 4 h p.i. in female NMRI mice treated with different compounds. (**A**) The kidney uptake of [^99m^Tc]Tc(CO)_3_-ADAPT6 in %ID/g of kidney. (**B**) The kidney uptake of [^99m^Tc]Tc(CO)_3_-ADAPT6 normalized to control taken as 100%. The uptake was decreased when 50 mmol/kg fructose or maleate were administered 5 min before the ADAPT6. Data are presented as average from four mice ± SD. An asterisk marks a significant difference from the control (* *p*  <  0.0001, one-way ANOVA).

Biodistribution of [^99m^Tc]Tc(CO)_3_-ADAPT6 was studied in female NMRI mice treated with various compounds 4 h p.i. of the radiolabeled protein. Sodium maleate, mannitol, furosemide, fructose, probenecid, or colchicine were injected to mice before the injection of [^99m^Tc]Tc(CO)_3_-ADAPT6 as described in Table 1. Lysine and gelofusine were administered as a co-injection together with [^99m^Tc]Tc(CO)_3_-ADAPT6. The control group received a single injection of [^99m^Tc]Tc(CO)_3_-ADAPT6. The biodistribution of [^99m^Tc]Tc(CO)_3_-ADAPT6 in all groups, except the group treated with 50 mmol/kg fructose, was similar to the control (*p*  >  0.05, one-way ANOVA test) (Table 2). Mice pre-injected with 50 mmol/kg fructose had higher retention of activity in blood, normal organs and carcass. However, in kidneys, the uptake of activity was reduced approximately 1.7 times (172  ±  27%ID/g) in comparison with the control (289  ±  47%ID/g) (Figure 2).

Another compound that caused a reduction of activity uptake in kidneys was sodium maleate. The activity was reduced 3.7-fold (78  ±  25% ID/g) compared with the control group (289  ±  47% ID/g) and, in the case of maleate, the reduction was observed only in kidneys (Figure 2). No significant (*p*  >  0.05, one-way ANOVA test) differences in activity uptake in other organs or tissues were observed.

**Figure 3:**
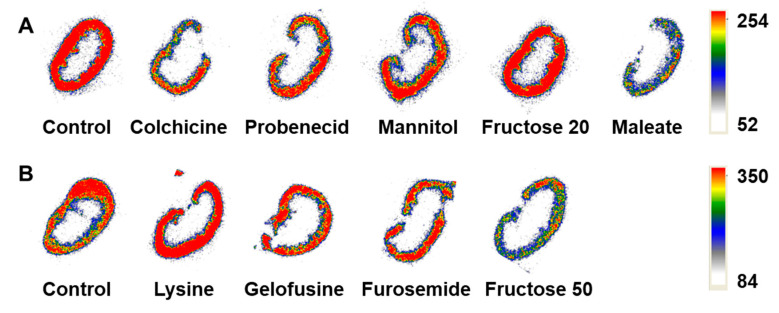
Representative ex vivo autoradiograms of the kidney sections of mice that were pre- or co-injected with (**A**) colchicine, probenecid, mannitol, fructose (20 mmol/kg), maleate or (**B**) lysine, gelofusine, furosemide, fructose (50 mmol/kg) before the injection of [^99m^Tc]Tc(CO)_3_-ADAPT6 in comparison with the control group that was injected with the radiolabeled protein only.

The average mouse weight was 28.0  ±  2.1 g, the average kidney weight was 280  ±  30 mg. The mice or kidney weights did not differ significantly (*p*  >  0.05, one-way ANOVA test) between the groups. After the gamma-spectrometer measurement, two pairs of kidneys from each group were used for autoradiography analysis. Representative autoradiograms of the kidney sections of mice injected with [^99m^Tc]Tc(CO)_3_-ADAPT6 (Figure 3) showed that the activity was localized in the kidney cortex in all studied groups.

## 3. Discussion

High accumulation of activity in kidneys is commonly observed for radiolabeled peptides, ESPs, and antibody fragments with size below ca. 60 kDa. These targeting agents have a short half-life in blood as they are efficiently filtered in the kidney glomeruli. Due to their proteinaceous nature, they are reabsorbed from primary urine in renal proximal tubuli and are degraded inside the lysosomes of cells. Fragments of degradation carrying radiolabels with residualizing properties (e.g., chelated radiometals) are retained in the cells leading to prolonged exposure of healthy tissues to radiation. The use of these targeting agents radiolabeled with residualizing labels for radionuclide therapy is mainly limited by high dose to kidneys.

The two receptors involved in the reabsorption of some peptides and small proteins in renal proximal tubules were identified as megalin and cubilin [35]. Studies in megalin-knockout mice models showed that megalin was involved in the reabsorption of [^111^In]In-DTPA-octreotide (ca. 70–85% reduction in kidney uptake compared with wild-type mice) [36], [^111^In]In-labeled octreotate, minigastrin, neurotensin and exendin (ca. 35–80% reduction) [37], and [^99m^Tc]Tc(CO)_3_-7C12 nanobody (ca. 40% reduction) [38]. However, megalin system was not involved in the reabsorption of affibody molecules [39].

One approach for reduction of renal reabsorption is based on the infusion of positively charged amino acids (lysine) or macromolecules containing charged residues (polyglutamic acid, succinilated gelatin (gelofusine)) that compete for binding to megalin/cubilin [40,41]. Some of these agents and their combination were found effective and are currently used during peptide receptor radionuclide therapy (PRRT) in clinics. Other factors that were proposed to influence renal accumulation of some radiopeptides were the number of charged amino acids in the peptide structure [42] and binding to different sites of endocytic receptors [41].

From the above-mentioned studies, it appears that the mechanism of reabsorption in kidneys and the factors influencing it could be unique for every class of radiolabeled targeting agents. In the current study, we investigated whether lysine and gelofusine, commonly used for PRRT in clinics, could reduce the renal uptake of ADAPTs. We also investigated other compounds that act on various parts of the reabsorption system in the kidney to better understand the mechanism behind the reuptake.

Maleate and colchicine were shown to change the distribution of megalin in the proximal tubules. Maleate inhibits protein reabsorption by lowering ATP production in the citric acid cycle in tubular cells [43,44]. Maleate dose in this study was chosen based on the study by de Jong et al., that showed the dose-dependent effect of sodium maleate for preventing reabsorption of [^111^In]In-DTPA-octreotide [45]. Sodium maleate effectively reduced renal uptake of somatostatin analogs to 15% of the uptake in control [32]. In the current study sodium maleate also significantly reduced the uptake of ADAPT6 to ca. 25% of the uptake in the control. However, its potential utility is limited by toxicity [46]. The effect of maleate on reduction of kidney uptake was more pronounced for ADAPTs than for affibody molecules or DARPins [33,34].

Colchicine inhibits polymerization of microtubules and interferes with intracellular transport of megalin from the cell membrane [44]. It was effective for reduction of kidney uptake of [^111^In]In-DTPA-octreotide, additionally supporting the hypothesis about the involvement of megalin in this process [30]. However, in the current study administration of the same dose of colchicine did not have any effect on kidney uptake of [^99m^Tc]Tc(CO)_3_-ADAPT6.

Probenecid modulates the transport of organic acids and acidic drugs by inhibiting organic anion transporters (OATs) in kidneys. It was reported to reduce the kidney uptake of [^111^In]In-DOTATOC by 30% [31]. The uptake of [^99m^Tc]Tc(CO)_3_-ADAPT6 was not influenced by probenecid which indicates that OATs are not involved in the reuptake of ADAPTs by the proximal tubular cells.

We also evaluated two types of diuretics, furosemide, and mannitol, that increase glomerular filter volume and flow rate. Renal activity accumulation of [^111^In]In-DOTATOC was increased by ca. 40% after furosemide administration [31]. However, the reabsorption of [^99m^Tc]Tc(CO)_3_-ADAPT6 was not influenced by furosemide or mannitol, which indicates that the reabsorption of ADAPT does not depend on filter volume or flow rate and suggests involvement of a high capacity reabsorption system.

Fructose is another compound that decreases ATP level in liver and kidneys when administered parenterally at high doses. A dose of 40 mmol/kg lowered ATP by 80% in the proximal straight tubule and by 60% in the proximal convoluted tubule in rat kidneys [47]. We have previously found that i.p. injection of 50–60 mmol/kg of fructose reduced the kidney uptake of radiolabeled affibody molecules and DARPins by ca. 50% [33,34]. As these doses are rather high and are close to the median lethal dose (LD_50_ 83 mmol/kg), we also included a lower dose of fructose (20 mmol/kg) in the study, however, no effect on reabsorption of [^99m^Tc]Tc(CO)_3_-ADAPT6 in kidneys was observed at a low dose. Administration of 50 mmol/kg fructose reduced the kidney uptake of [^99m^Tc]Tc(CO)_3_-ADAPT6 by ca. 60%. An increased retention of activity [^99m^Tc]Tc(CO)_3_-ADAPT6 in blood and normal organs most likely indicate the impairment of its secretion by kidneys.

The findings obtained for the three studied classes of ESPs, affibody molecules, DARPins and ADAPTs follow a common trend. Both maleate and fructose reduce the kidney uptake of [^99m^Tc]Tc(CO)_3_-ADAPT6. Cortical accumulation of activity seen on autoradiography supports the involvement of a proximal tubular mechanism in the reabsorption of [^99m^Tc]Tc(CO)_3_-ADAPT6, similar to other targeting radiolabeled peptides and proteins.

## 4. Material and Methods

### 4.1. General

Technetium-99m was eluted as pertechnetate from the Ultra-TechneKow generator (Mallinckrodt, Petten, The Netherlands) with sterile 0.9% sodium chloride. The CRS (Center for Radiopharmaceutical Sciences) kits for the production of tricarbonyl technetium were purchased from the CRS (PSI, Switzerland). Sodium maleate, d-fructose, colchicine, probenecid, and l-lysine were purchased from Sigma (Sigma-Aldrich, St Louis, MO, USA). Furosemide (Takeda Pharma AB, Sweden), mannitol (Fresenius Kabi AB, Sweden), and gelofusine (B. Braun, Melsungen, Germany) were purchased as solutions for injections. The anti-HER2 ABD-derived affinity protein (ADAPT6) was produced in *E. coli* strain BL21(DE3) as described previously [12]. ADAPT6 has a hexahistidine tag at N-terminus for site-specific labeling with tricarbonyl technetium. TLC analysis was performed using iTLC silica gel strips (Varian, Lake Forest, CA, USA). The radioactivity distribution along iTLC strips was measured using a Cyclone phosphor system and analyzed by the OptiQuant image analysis software (both from PerkinElmer, Waltham, MA, USA). To cross-validate radio-iTLC data, radio-high performance liquid chromatography (HPLC) analysis was performed using reversed-phase HPLC. Hitachi Chromaster HPLC systems with radioactivity detector and Vydac RP C18 column (300 Å; 3 × 150 mm; 5 μm) at room temperature was used. Solvent A was 0.1% trifluoroacetic acid (TFA) in H_2_O; solvent B was 0.1% TFA in acetonitrile. The flow rate was 1 mL/min, with a 5% B to 80% B gradient over 20 min. Radioactivity in organs and tissues was measured using an automated gamma-spectrometer with a NaI (TI) detector (1480 Wizard, Wallac, Finland).

### 4.2. Radiolabeling of ADAPT6

Site-specific radiolabeling of hexahistidine-containing ADAPT6 with [[^99m^Tc]Tc(CO)_3_]^+^ to obtain a residualizing label was performed as described earlier [7,15]. Purification of the radiolabeled ADAPT6 by size-exclusion chromatography was performed using NAP-5 columns (GE Healthcare, Amersham, UK) pre-equilibrated and eluted with PBS. Radiochemical yield and purity were measured using radio-iTLC in PBS. The purity of the label was cross-validated using RP-HPLC as described above.

### 4.3. Animal Studies

All handling of animals had been approved by the Ethics Committee for Animal Research in Uppsala County (Permit Number: C4/ 2016) and was performed according to the Swedish laws on laboratory animal welfare. Female NMRI mice (10 weeks old; Scanbur A/S) were housed in standard conditions at 22 °C, with laboratory food and water provided ad libitum. Mice had an adaptation period of one week before the start of experiments. For biodistribution studies, forty mice were randomized in ten groups to include four animals per group. To investigate the effect of compounds on the kidney uptake of [^99m^Tc]Tc-labeled ADAPT6, mice were treated with one compound per group according to Table 2 before administration of [^99m^Tc]Tc(CO)_3_-ADAPT6, except lysine and gelofusine, which were co-administered with [^99m^Tc]Tc(CO)_3_-ADAPT6. Radiolabeled ADAPT6 (60 kBq; 0.42 nmol, 3.0 μg) in 100 μL of 1% BSA in PBS per mouse was administered intravenously (i.v.). The total protein amount was adjusted using non-labeled ADAPT6. Four hours after injection of [^99m^Tc]Tc-labeled ADAPT6, mice were anesthetized by an intraperitoneal (i.p.) injection of ketamine and xylazine and sacrificed by heart puncture. Blood and organs were collected, weighed, and activity was measured using a gamma-spectrometer. The data was corrected for decay, and percent of injected dose (ID) per gram of sample was calculated, except for GI tract and carcass, where %ID per whole sample was calculated.

**Table 2:** List of compounds, their proposed mechanism of action and administration protocol preceding the injection of [^99m^Tc]Tc(CO)_3_-ADAPT6.

Compound	Proposed Mechanism of Action in the Kidney	Administration Route	Administration Respective to the [^99m^Tc]Tc(CO)_3_-ADAPT6	Dose	LD_50_
Lysine	Blocks megalin ligand-binding domains [48]	i.v.	Co-injection	1200 mg/kg	4000 mg/kg (i.p., rat)
Gelofusine	Blocks megalin ligand-binding domains [49]	i.v.	Co-injection	160 mg/kg	n/a
Sodium maleate	Inhibits ATP-mediated endocytosis [44,45,50]	i.v.	5 min before	480 mg/kg	600 mg/kg (i.p., rat) 3380 mg/kg (oral, rat)
Mannitol	Osmotic diuretic, reduces contact time with scavenger receptors [31]	i.v.	5 min before	480 mg/kg	7470 mg/kg (i.v., mouse)
Furosemide	Diuretic, reduces contact time with scavenger receptors [31]	i.v.	5 min before	3 mg/kg	800 mg/kg (i.p., rats)
Fructose	Inhibits ATP-mediated endocytosis [47]	i.p.	5 min before	3.6 and 9 g/kg (20 and 50 mmol/kg)	15 g/kg (83 mmol/kg)
Probenecid	Inhibits organic anion transporter and reduces renal excretion of drugs [31]	i.p.	1 h before	24 mg/kg	1000 mg/kg (i.p., mouse)
Colchicine	Inhibits polymerization of microtubules and recycling of megalin [51]	i.p.	5 h before	1.2 mg/kg	1.6 mg/kg (i.p., mouse)

### 4.4. Autoradiography

Autoradiography of kidneys was performed as described previously [34]. The kidneys were embedded in the frozen section medium (Richard-Allan Scientific Neg-50, Thermo Fisher Scientific, Kalamazoo, MI, USA), frozen at −80 °C, cut in serial sections (30 μm thick) using a cryomicrotome (CryoStar NX70, Thermo Fisher Scientific, Runcorn, UK), and thaw-mounted on glass slides. The slides with sections were put in an X-ray cassette and exposed to phosphor screens overnight. The screens were scanned using a Cyclone Storage Phosphor System at a resolution of 600 dpi and analyzed using the OptiQuant software.

### 4.5. Statistical Analysis

Statistical analysis was performed using GraphPad Prism (Prism 8 for Windows, GraphPad Software, San Diego, CA, USA). One-way ANOVA test with Bonferroni correction for multiple comparisons was used to find significant differences.

## 5. Conclusions

The agents, commonly used for PRRT in clinics were not effective for reduction of kidney uptake of [^99m^Tc]Tc(CO)_3_-ADAPT6. The reduction of uptake by fructose is only achieved at high doses similarly to maleate, and the potential utility of these agents is limited by their toxicity. Therefore, other strategies for reduction of kidney uptake, such as pretargeting or the use of cleavable linkers, appear more promising and could be further explored in order to use ADAPTs for targeted radionuclide therapy.

## Figures and Tables

**Table 1:** Biodistribution of [^99m^Tc]Tc(CO)_3_-ADAPT6 in NMRI mice 4 h p.i. administered alone (control) or after treatment with the compounds.

	Blood	Salivary Glands	Liver	Spleen	GI Tract	Carcass
Control	0.19 ± 0.02	0.15 ± 0.02	1.2 ± 0.1	0.47 ± 0.04	1.0 ± 0.1	1.8 ± 1.3
Lysine	0.10 ± 0.01	0.10 ± 0.02	1.5 ± 0.1	0.7 ± 0.2	1.2 ± 0.5	1.4 ± 0.2
Gelofusine	0.09 ± 0.01	0.11 ± 0.01	1.3 ± 0.1	0.3 ± 0.1	1.1 ± 0.6	1.3 ± 0.1
Mannitol	0.21 ± 0.02	0.15 ± 0.06	1.1 ± 0.2	0.6 ± 0.1	1.3 ± 0.2	2.0 ± 0.7
Furosemide	0.12 ± 0.02	0.14 ± 0.02	1.2 ± 0.1	0.5 ± 0.1	1.3 ± 0.5	1.8 ± 0.2
Probenecid	0.23 ± 0.03	0.13 ± 0.02	1.1 ± 0.1	0.6 ± 0.1	0.9 ± 0.1	1.6 ± 0.2
Colchicine	0.25 ± 0.03	0.12 ± 0.05	1.0 ± 0.3	0.7 ± 0.2	0.8 ± 0.3	1.7 ± 0.4
Fructose 20 mmol/kg	0.25 ± 0.02	0.21 ± 0.03	1.4 ± 0.2	0.5 ± 0.1	1.4 ± 0.1	2.5 ± 0.3
Fructose 50 mmol/kg	2.0 ± 1.0 *	0.7 ± 0.4 *	4.1 ± 1.1 *	1.7 ± 0.5 *	5.4 ± 1.6 *	14.4 ± 6.6 *
Maleate	0.13 ± 0.06	0.11 ± 0.03	1.1 ± 0.2	0.5 ± 0.1	0.9 ± 0.3	1.4 ± 0.4

Data are presented as average from four mice ±  standard deviation (SD). Uptake is shown in %ID/g, except for GI tract and carcass, which is shown in %ID per whole sample. An asterisk marks a significant difference between the control and the treated group (* *p*  <  0.0001, one-way ANOVA test).
